# Genome-wide analysis of DNA Methylation profiles on sheep ovaries associated with prolificacy using whole-genome Bisulfite sequencing

**DOI:** 10.1186/s12864-017-4068-9

**Published:** 2017-10-02

**Authors:** Yanli Zhang, Fengzhe Li, Xu Feng, Hua Yang, Aoxiang Zhu, Jing Pang, Le Han, Tingting Zhang, Xiaolei Yao, Feng Wang

**Affiliations:** Jiangsu Engineering Technology Research Center of Mutton Sheep and Goat Industry, College of Animal Science and Technology, Nanjing Agricultural University, No.1 Weigang, Nanjing, Jiangsu 210095 China

**Keywords:** DNA Methylation, WGBS, Prolificacy, Ovary, Sheep

## Abstract

**Background:**

Ovulation rate and litter size are important reproductive traits in sheep with high economic value. Recent work has revealed a potential link between DNA methylation and prolificacy. However, a genome-wide study that sought to identify potential DNA methylation sites involved in sheep prolificacy indicated that it is still unknown. Here, we aimed to investigate the genome-wide DNA methylation profiles of Hu sheep ovaries by comparing a high-prolificacy group (HP, litter size of three for at least 2 consecutive lambings) and low prolificacy group (LP, litter size of one for at least 2 consecutive lambings) using deep whole-genome bisulfite sequencing (WGBS).

**Results:**

First, our results demonstrated lower expression levels of DNA methyltransferase (*DNMT*) genes in the ovaries of the HP group than that in the ovaries of the LP group. Both groups showed similar proportions of methylation at CpG sites but different proportions at non-CpG sites. Subsequently, we identified 70,899 differential methylated regions (DMRs) of CG, 16 DMRs of CHG, 356 DMRs of CHH and 12,832 DMR-related genes(DMGs). Gene Ontology (GO) analyses revealed that some DMGs were involved in regulating female gonad development and ovarian follicle development. Finally, we found that 10 DMGs, including *BMP7*, *BMPR1B*, *CTNNB1*, *FST*, *FSHR*, *LHCGR*, *TGFB2* and *TGFB3,* are more likely to be involved in prolificacy of Hu sheep, as assessed by correlation analysis and listed in detail.

**Conclusions:**

This study revealed the global DNA methylation pattern of sheep ovaries associated with high and low prolificacy groups, which may contribute to a better understanding of the epigenetic regulation of sheep reproductive capacity.

**Electronic supplementary material:**

The online version of this article (doi:10.1186/s12864-017-4068-9) contains supplementary material, which is available to authorized users.

## Background

DNA methylation is an epigenetic regulatory mechanism that plays a significant role in mediating biological processes such as gene expression, genomic imprinting, cell differentiation and embryogenesis, as well as in determining phenotypic plasticity in organisms [1]. DNA methylation occurs at the cytosine residue of CpG dinucleotides, which are unevenly distributed throughout the genomes. Recently, the whole genome methylation of genes involved in vital biological functions has been extensively examined in mammalian species [2, 3] by using advanced high-throughput sequencing technologies. The reproductive efficiency of sheep in terms of litter size has an important impact on the economic returns of farmers [4]. Reproductive traits typically have low to medium heritability and do not exhibit a noticeable response to phenotypic selection [4]. Therefore, investigation of the genetic information associated with reproductive ability could efficiently enhance selection. Prolificacy is controlled by ovarian folliculogenesis, a process that is highly regulated by precise proliferation and differentiation events. Recent research has shifted focus to how DNA methylation regulates the initiation of ovarian and sexual maturation. Evidence has revealed that ovarian maturation is regulated by DNA methylation [5, 6]. By profiling the methylome of porcine ovaries, researchers examined the methylation changes during the process of sexual and ovarian maturation in pigs [7]. Similar studies also found that DNA methylation alterations influenced gene expression profiles in the goat hypothalamus during the onset of puberty [8]. Despite these findings, our understanding of DNA methylation patterns associated with prolificacy remains limited. Hu sheep are widely recognized as having early sexual maturity and high prolificacy; however, in recent years, much attention has been focused on meat rather than reproductive traits during the selection process. Reproduction is a complex process, and traits such as litter size are affected by many minor genes and some major genes; thus, understanding the role of DNA methylation in gene function is necessary.

Of the four DNA methylation sequencing technologies: methylated DNA binding domain sequencing, methylated DNA immunoprecipitation sequencing, representation bisulfite sequencing (RRBS) and whole-genome bisulfite sequencing (WGBS), WGBS is similar to whole genome sequencing, except for one detail: bisulfite conversion. It achieves single-base resolution through bisulfite conversion and does not have a base preference as it can yield almost complete information about methylcytosine with excellent specificity and non-sensitivity [9]. Therefore, WGBS is the most comprehensive of the existing methods. In this study, we investigated DNA methylation profiles in the ovaries of high and low prolificacy sheep at 3 years of age during the estrus stage using WGBS technology. Our research systematically analyzed the DNA methylation patterns potentially involved in litter size. In addition, our findings would advance knowledge and understanding of the sheep methylome.

## Methods

### Animals and tissue collection

A total of 6 non-pregnant ewes with identical lambing records (3 records) were selected and divided into a high prolificacy group (HP: *n* = 3, litter size = 3) and a low prolificacy group (LP: *n* = 3, litter size = 1). Progesterone sponges were intravaginally implanted in ewes for estrus synchronization and were removed after 11 days. Then the estrus status of the ewes was checked every day. Ewes were slaughtered within 12 h during the estrus stage, and ovaries were immediately collected and snap-frozen in liquid nitrogen immediately and then stored at −80 °C until use.

### DNA, bisulfite treatment and RNA/cDNA preparation

Each whole ipsilateral ovary per sheep was collected, and the genomic DNA was extracted using a Genomic DNA kit (TIANamp, Cat.#DP304–02), and then bisulfite conversion was performed using the EZ DNA Methylation Direct Kit (Zymo Research Corporation, Cat#D5020). Total RNA was isolated using the TRIzol reagent (Invitrogen Corp, Cat.#15,596–026) and dissolved in RNase-Free water (QIAGEN, Cat.#129,112). The quality and quantity of DNA and RNA were determined using a NanoDrop instrument (NanoDrop Technologies, Wilmington, DE, USA). cDNA samples were synthesized from total RNA using a reverse transcription (RT) reagent kit with gDNA Eraser (Takara, Cat.#RR047A). All operations were conducted following the manufacturer’s recommended instructions.

### WGBS library preparation and data analysis

Three samples from each group were selected for WGBS sequencing. Genomic DNA was fragmented by ultrasonication. The fragments were then end-repaired, 3′-end-adenylated and ligated with adapters. Agarose gel electrophoresis was used to select fragments of 400–500 bp in length. The selected fragments were treated with bisulfite and subjected to PCR amplification to form the sequencing library. Then, the qualified library was sequenced using an IlluminaHiSeq™2500 system (Biomarker Technologies, Beijing, China). The peak signal produced by the Illumina HiSeq was transformed into base sequence by base calling as Raw Data or Raw Reads. The Raw Reads were then filtered for subsequent information analysis to ensure the quality of information analysis, including the removal of reads that have adapters and filtration of reads with more than 10% N content or more than 50% low quality bases. The final filtered data are called clean reads.

### Mapping reads to known genome

The sequencing reads need to be aligned with the reference genome (Oar_v3.1) before conducting the methylation analysis. Bismark software was used to perform a comparison of the alignments of bisulfite-treated reads to a reference genome using the default parameters. Reads that aligned with the same region of the genome were taken as the duplicate number. And the duplicate number was used to summarize the sequencing depth and coverage. The conversion rate of bisulfite was calculated as the percentage of the methylated clean reads as a percentage of the total number of clean reads in the lambda genome by using Bismark software. As unmethylated cytosine from the genome was converted into T after bisulfite treatment and PCR amplification, but methylated cytosine remained unchanged. Bismark was able to extract information about genome cytosine sites from the results of the comparison of the clean reads with the reference genome and thereby acquire cytosine site coverage statistics and the number of different types (CG as CpG, CHG and CHH) of methylated cytosine reads. As the methylation single C site cannot be discriminated by Bismark, we used the binomial distribution test for each C site to confirm the methylated C site by screening conditions for coverage ≥4× and false discovery rate (FDR) < 0.05.

### Estimating methylation levels and the identification of DMRs

All cytosine sites with reads coverage >10X were used for DMRs analysis with MOABS [10]. First, to detect the different methylated C sites in a region, we defined *C*_*i*_ as the number of supporting methylation reads at a single C site, *T*_*i*_ as the number of supporting unmethylation reads at a single C site, *i* as the position of C, and *n* as the total number of C positions. The methylation level of a C site was counted as follows [11]:

$$ \mathrm{Methylation}\  \mathrm{level}\  \mathrm{of}\ \mathrm{C}\ \mathrm{site}={C}_i/\left({C}_i+{T}_i\right) $$

The binomial distribution test was used to determine whether the C site was methylated. Subsequently, DMRs were defined that three different methylation sites at least in the region, and in which the difference in methylation levels was greater than 0.2 (0.3 for CG type) with *p* value from Fisher’s exact test of less than 0.05. The methylation level of regions was counted as follows [11]:

$$ \mathrm{Methylation}\  \mathrm{level}\  \mathrm{of}\  \mathrm{region}=\frac{1}{n}\times \sum_{i=1}^n\frac{C_i}{C_i+{T}_i} $$

### Bioinformatics analysis of DMGs

The DMGs were compared with functional databases such as GO, COG (Cluster of Orthologous Groups of proteins) and KEGG (Kyoto Encyclopedia of Genes and Genomes) by BLAST to obtain the annotation of these genes for analyzing gene function. The GO enrichment analysis was implemented by the Wallenius non-central hyper-geometric distribution in the GOseq R packages [12]. KOBAS software was used to test for statistically significant enrichment of differentially expressed genes in KEGG pathways [13]. The interaction networks of selected DMGs were analyzed using the String database (http://string-db.org/) [14].

### Quantitative reverse transcriptase PCR

Quantitative reverse transcriptase PCR (qRT-PCR) was used to examine DNA methyltransferase gene expression levels and validate the DMGs from the sequencing results by detecting the mRNAs expression level. Ten DMGs were randomly selected, and the specific primers used in the qRT-PCR are listed in Additional file 1: Table S1.

qRT-PCR was performed on a StepOnePlus Real-Time PCR System (Life Technologies, USA). Each PCR (a reaction volume of 20 μL) system included 10 μL of SYBR Green Master mix (Roche Applied Science, Mannheim, Germany), 0.6 μL each of 10 μM forward and reverse primer, 1 μL of reverse transcription product and 7.8 μL of RNase-Free water. The comparative quantification of each of the results was standardized to *GAPDH* by the 2^-ΔΔCt^ method.

### Statistical analysis

All data were analyzed using the SPSS 24.0 software package (SPSS, Chicago, IL, USA). The qRT-PCR results are expressed as the mean ± standard error, and each group contained three samples and the experiments were repeated at least 3 times. The different mRNA expression levels of genes in the HP and LP groups were compared using the independent-samples *t*-test. Differences were considered significant at *P* < 0.05.

## Results

### Expression levels of DNMTs

**Fig. 1:**
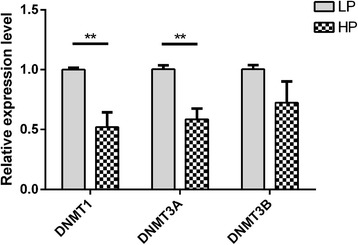
The mRNA expression level of DNMTs as determinded by qRT-PCR. The relative expressions of DNMTs in ovaries was detected by qRT-PCR. The experiment was performed using 3 biological repeats and 3 technical repeats. The relative expression levels were normalized to the expression amount of GAPDH. The results are expressed relative to the LP group as the mean ± SEM and the ordinate represents log10-transformed values. **, *P* < 0.01

The expression levels of *DNMTs* (*DNMT1, DNMT3A and DNMT3B*) in ovaries were first analyzed by qRT-PCR in the HP and LP sheep. As Fig. 1 shows, *DNMT1* and *DNMT3A* were expressed at significantly lower levels in the HP group than in the LP group (*P* < 0.05).

### DNA methylation mapping and patterns

A total of 63.79 G and 66.72 G raw bases were generated on average for the HP group and the LP group respectively. After data filtering, approximately 200 million clean reads were generated for each group. These reads were detected in all chromosomal regions for each group. The mapped reads were used for subsequent analysis as the rates were from 71.36% to 74.68% (Table 1).

**Table 1:** Whole genome DNA bisulfite sequencing data

Groups	Sample	Clean Base (Gb)	Clean Reads	Mapped (%)	Bisulfite Conversion Rate (%)	Total_mC (%)
HP	J07	66.06	221,116,974	73.88	99.35	3.65
	J08	62.23	207,777,912	74.31	99.36	3.61
	J09	62.47	208,906,017	74.53	99.38	3.53
LP	J10	63.61	212,805,686	74.68	99.39	3.49
	J11	71.87	240,143,585	73.74	99.49	3.40
	J12	64.68	216,081,063	71.36	99.43	3.62

*Clean Base (Gb)* The number of Clean Base throughout the sequence

*Clean Reads* The number of clean reads

*Mapped (%)* The number of clean reads matched to the reference genome relative to the total clean reads

*Bisulfite Conversion Rate (%)* The number of clean reads matched to the reference genome relative to the amount of methylation of the clean reads matched to the reference genome

*Total mC (%)* The number of clean reads matched to the reference genome relative to the amount of methylated cytosine within the clean reads matched to the reference genome

**Fig. 2:**
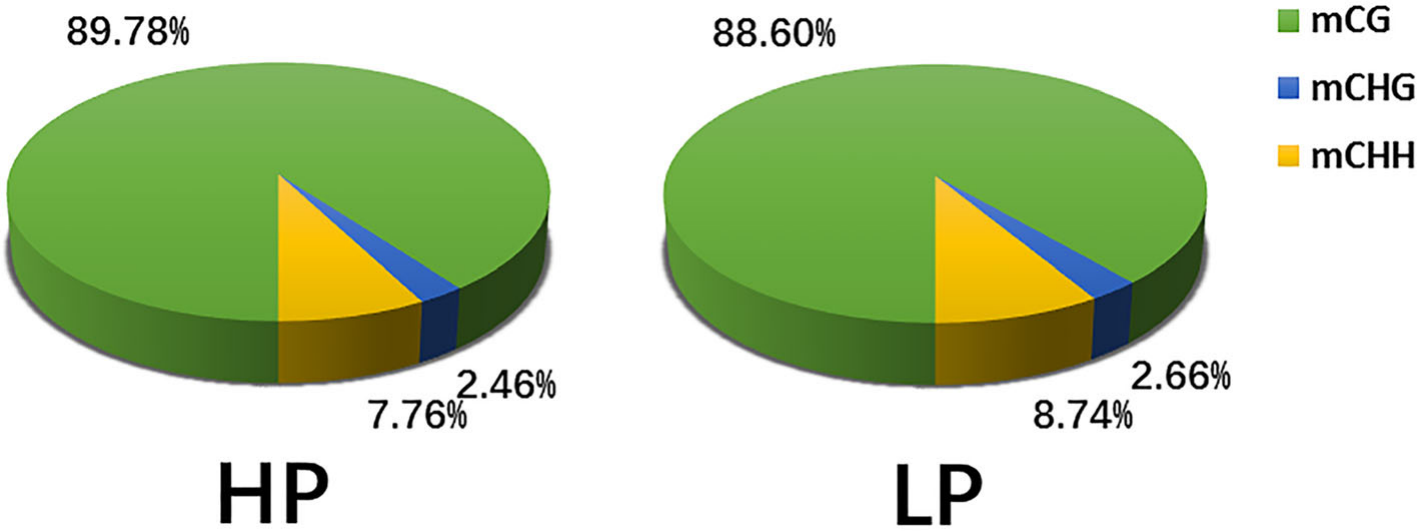
The average ratio of DNA methylation types in the genomes of HP and LP. HP, High Prolificacy. LP, Low Prolificacy, H = A, C or T. The green, blue and yellow colors represent mCG, mCHG and mCHH, respectively

In each group, approximately 3.5% of all genomic C sites were methylated (Table 1). Methylation in sheep was found to exist in three sequence contexts: CG, CHG (where H is A, C or T), and CHH. These contexts were present in similar proportions in each group, and we found overall genome-wide methylated cytosine levels of 89.78% CG, 2.46% CHG, 7.76% CHH in the HP group and 88.60% CG, 2.66% CHG, 8.74% CHH in the LP group (Fig. 2).

### Sequence preferences analysis for methylation

**Fig. 3:**
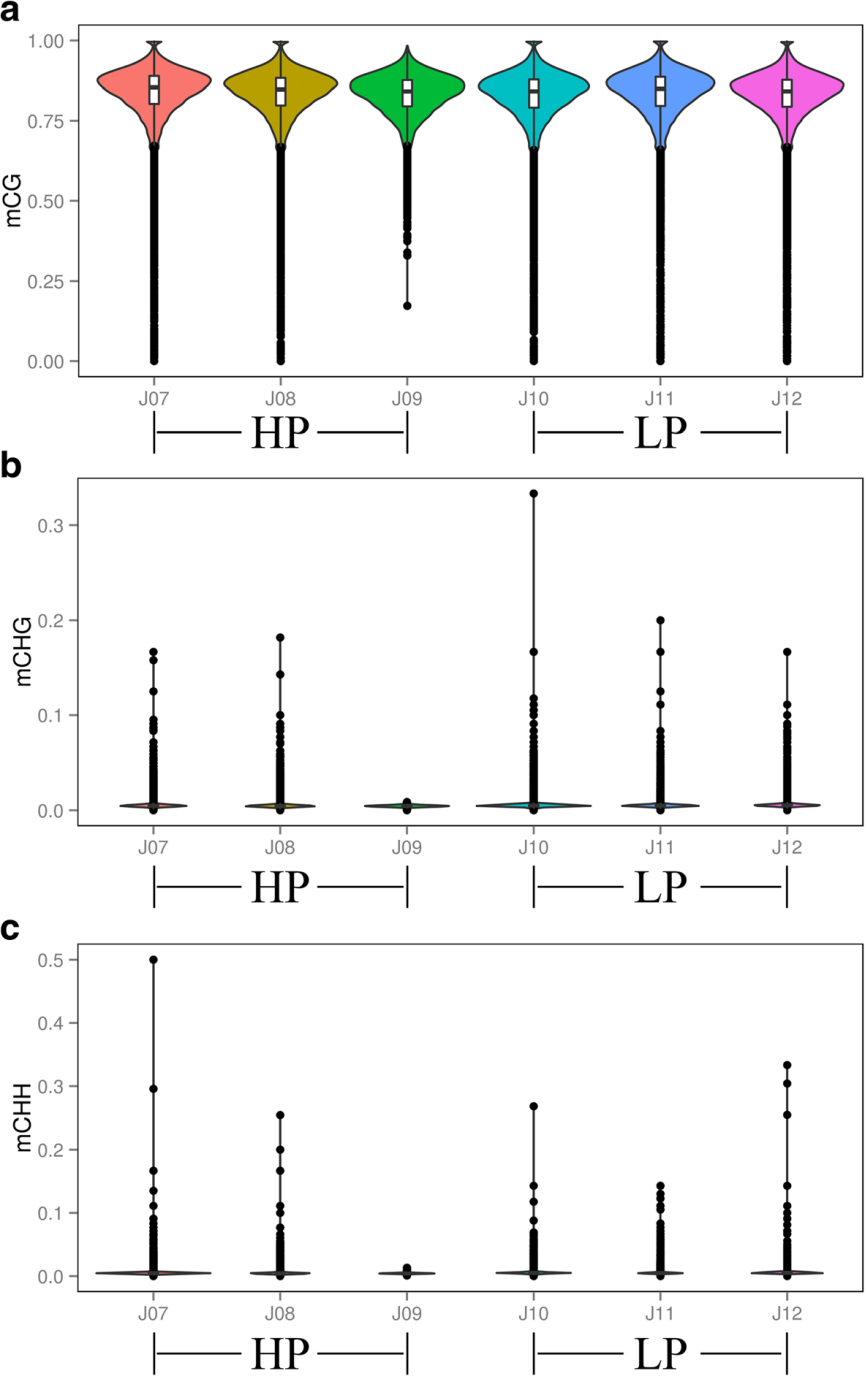
Violin plot for the overall distribution of methylation levels for different methylation types. a, CG. b, CHG. c, CHH. HP (J07, J08, J09), High Prolificacy. LP (J10, J11, J12), Low Prolificacy. H = A, C or T. The abscissa represents the different samples, the ordinate represents the level of methylation of the samples; and the width of each violin represents the density of the point at that methylation level; while the boxplot shows the methylation levels in each violin

A violin graph was plotted with dots representing different methylation levels, and we found that the methylation levels were high with wide sections in the violin plot for CG methylation types (Fig. 3a), but the methylation levels were low with narrow sections in the violin plot for CHG and CHH methylation types (Fig. 3b and c). Then, we plotted chromosome methylation maps for each sample. The results showed that most hypermethylation cytosine were of the CG type in chromosomes and that the mC (methylated cytosine) sites were different on the chromosome like chromosome 18 between the two groups (Additional file 2: Figure S1).

**Fig. 4:**
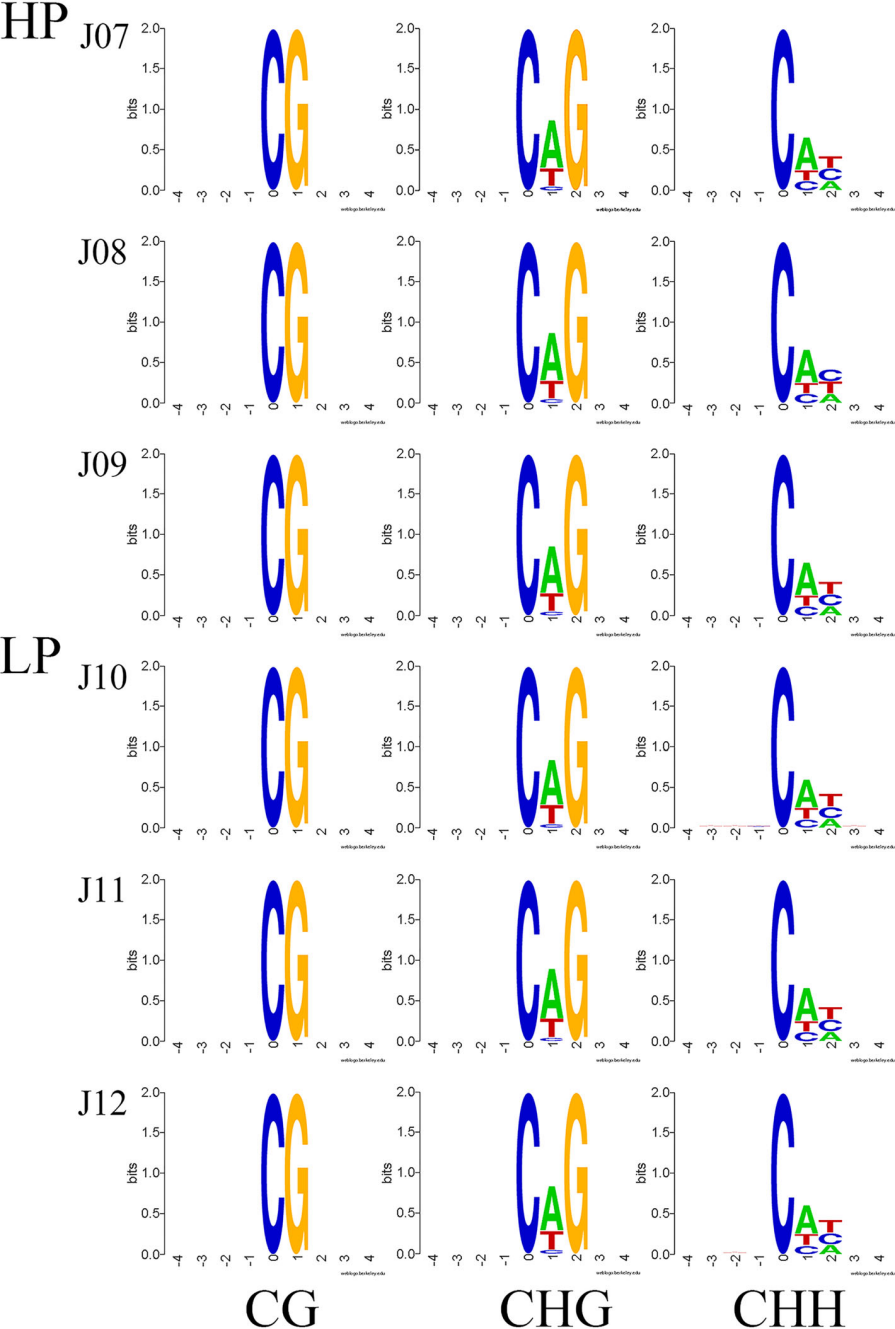
Methylation preferences in 9 bp spanning CG, CHG, and CHH methylcytosine sites. HP (J07, J08, J09), High Prolificacy. LP (J10, J11, J12), Low Prolificacy. H = A, C or T. The abscissa is the base number of the methylation site, the total height of each position is the sequence conservation of the base, which represents the relative frequency of the base at that position

Furthermore, we analyzed the relationship between sequence context and methylation preference. We calculated the percentage methylation for all possible 9-mer sequences in which either the methylated cytosine was in the fourth position (allowing analysis of the three nucleotides upstream of CG, CHG, and CHH methylation), the mC sites were in a hypermethylation state, the CAG was the most common sequence motif in the CHG mC sites, and different frequencies of the CHH contexts was discovered for the two groups (Fig. 4).

### DNA methylation levels of different functional regions

**Fig. 5:**
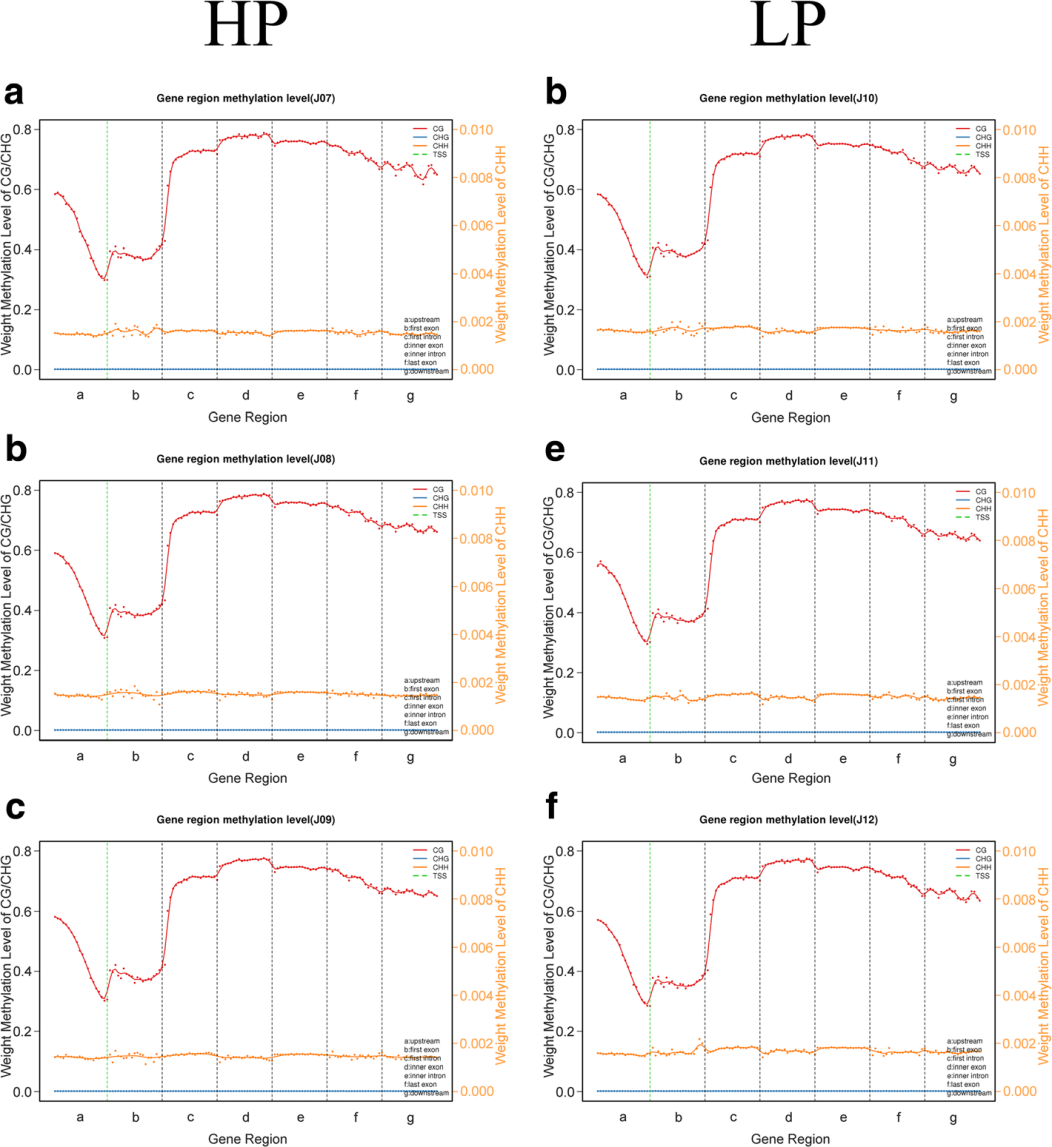
The trend in CGI distribution in different functional elements. **a**, J07. **b**, J08. **c**, J09. **d**, J10. **e**, J11. **f**, J12. HP (J07, J08, J09), High Prolificacy. LP (J10, J11, J12), Low Prolificacy. H = A, C or T. The abscissa represents the different regions of gene functional elements that a, b, c, d, e, f, g denote upstream, first exon, first intron, inner exon, inner intron, last exon and downstream, respectively. The left ordinate represents the methylation levels of CG/CHG, and the right ordinate represents the methylation levels of CHH. The dotted, green, vertical line represents the TSS, and the red, orange and blue solid lines represent CG, CHH and CHG, respectively, which show the methylation levels fluctuating in the different regions

We divided all mC into specific gene features: promoter, 5’UTR (Untranslated Regions), exons, introns, and 3’UTR. The methylation levels were evaluated in these functional regions. As shown in Fig. 5, the trend of methylation levels in the specified regions of the two groups were similar in the two groups, and the methylation levels for the CG type were higher than those for the CHG and CHH types. Intron regions, exons (except for the first exon) and downstream regions are the major components of mC containing sites (Additional file 3: Table S2). Moreover, the methylation levels of CG in the first exon were lower than those of the other elements except for the upstream region, as the levels showed a downward trend in upstream region, and the methylation levels of CG sites near the TSS were lower than those in the first exon. In addition, high levels of DNA methylation were detected in inner exons and introns, and the levels of methylation decreased gradually from the promoters to the TSS (Transcription Start Site) and increased from the TSS to the introns; the CHH type was hypomethylation and stable in each functional element; the CHG type was almost entirely unmethylated. More detailed information is listed in Additional file 4: Table S3.

### Annotation of methylation CGI regions

**Fig. 6:**
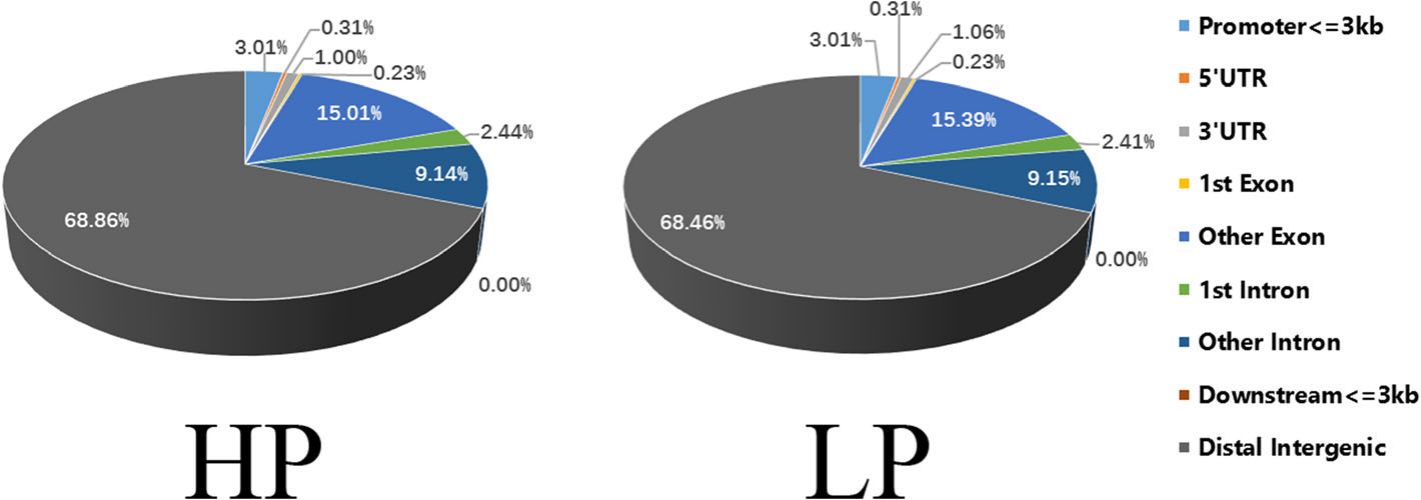
The ratio of hypermethylation CGI distribution in different functional elements of gene. HP, High Prolificacy. LP, Low Prolificacy. The charcoal, blue, orange, gray, yellow, hyacinthine, green, dark blue and brown colors represent distal intergenic, promoter (<=3 kb), 5’UTR, 3’UTR, 1st exon, other exon, 1st intron, other intron and downstream regions (<=3 kb), respectively

We counted the quantity of hypermethylation CGI (CG islands) regions (hypermethylation CGI definition: methylation level over 0.7 except when the proportion of C sites with high confidence was less than 0.1) and annotated these with gene functional elements (with 3000 bp 5′ to the TSS and 3′ to transcription termination site as the gene upstream and downstream functional regions respectively). As Fig. 6 shows, approximately 68% hypermethylation CGI was distributed in distal intergenic regions, within 1.5% hypermethylation CGI was distributed in UTR and there was no significant difference between the two groups (*P* > 0.05).

### DMRs analysis for the HP and LP groups

**Fig. 7:**
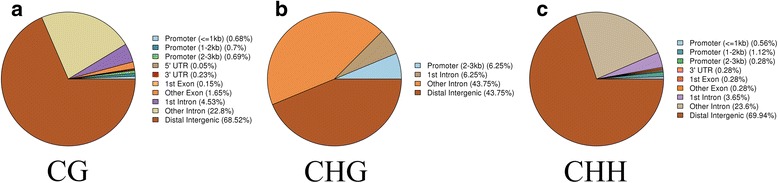
The ratio of DMRs with different methylation types in different gene functional regions. a, CG. b, CHG. c, CHH. H = A, C or T. The blue, dark blue, green, khaki, red, yellow, orange, purple, light yellow and brown colors represent promoter (<=1 kb), promoter (1-2 kb), promoter (2-3 kb),5’UTR, 3’UTR, 1st exon, other exon, 1st intron, other intron and distal intergenic regions, respectively

**Fig. 8:**
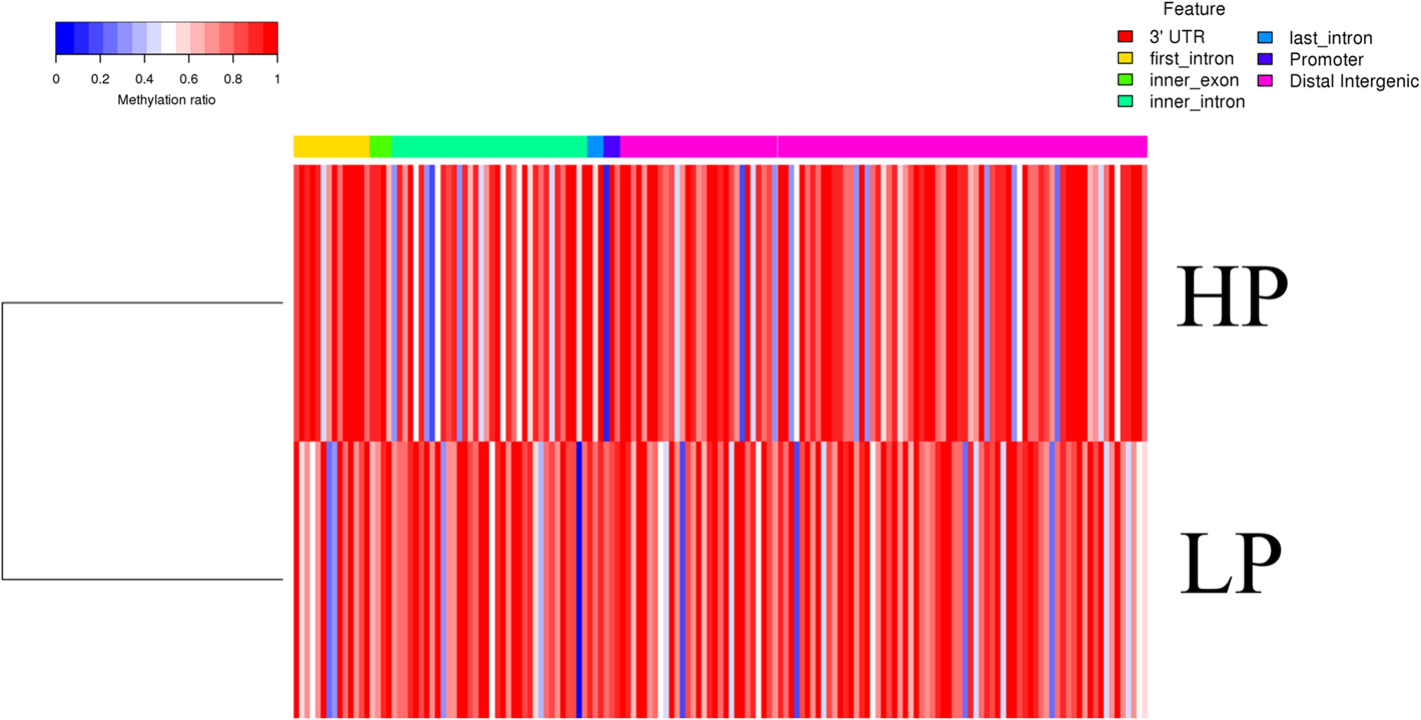
Heat map cluster analysis of DMRs in different gene functional regions. Each column represents an individual DMR and each row represents one group. The colors in each block from blue to white to red sequentially represents the methylation ratio from 0 to 0.5 to 1, respectively. In addition, the red, yellow, green, turquoise, blue, purple and pink colors represent the 3’UTR, first intron, inner exon, inner intron, last intron, promoter and distal intergenic regions respectively which are shown above the heatmap

DMRs were detected in the two groups and were annotated into gene functional elements according to different methylation types. In total, 70,899 CG DMRs, 16 CHG DMRs and 356 CHH DMRs were identified, most of which were in distal intergenic regions, with only 33 and 162 DMRs were in the 5’UTR and 3’UTR respectively. For all methylation types, the ratio of DMRs located in introns was the highest except for those in distal intergenic regions (Fig. 7). The difference in methylation levels between the two groups in chr12(74369711–74,369,728), chr14(9182359–9,182,369), chr12(69412005–69,412,018), chr3(176960930–176,960,977) and chr1(230630159–230,630,190) had exceed 90% (Additional file 5: Table S4). A heat map was generated using a cluster analysis of DMRs for the HP and LP groups (Fig. 8). More detailed DMRs results are listed in Additional file 5: Table S4.

### Verification of sequencing results

**Fig. 9:**
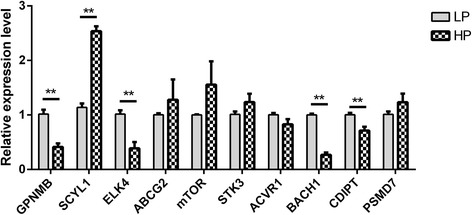
The mRNA expression level of DMGs as determined by qRT-PCR. The relative expressions of DMGs in ovaries was detected by qRT-PCR. The experiment was performed using 3 biological repeats and 3 technical repeats. The relative expression levels were normalized to the expression amount of GAPDH. The results are expressed relative to the LP group as the mean ± SEM, and the ordinate represents log10-transformed values. *, *P* < 0.05. **, *P* < 0.01

To further validate the sequencing results, 10 DMGs from the sequencing results were randomly selected for detection by qRT-PCR. As shows in Fig. 9, the *GPNMB*, *ELK4*, *BACH1*, *CDIPT* levels were significantly lower and the *SCYL1* levels were significantly higher in the HP than in the LP group (*P* < 0.05), and the *ABCG2*, *mTOR*, *STK3*, *ACVR1* and *PSMD7* levels were not significantly different between the two group (*P* > 0.05). More detailed information of these genes/DMRs is listed in Additional file 6: Table S5. In total, the qRT-PCR results showed that the sequencing data were reliable.

### Database enrichment analysis for DMGs: COG, GO and KEGG

**Fig. 10:**
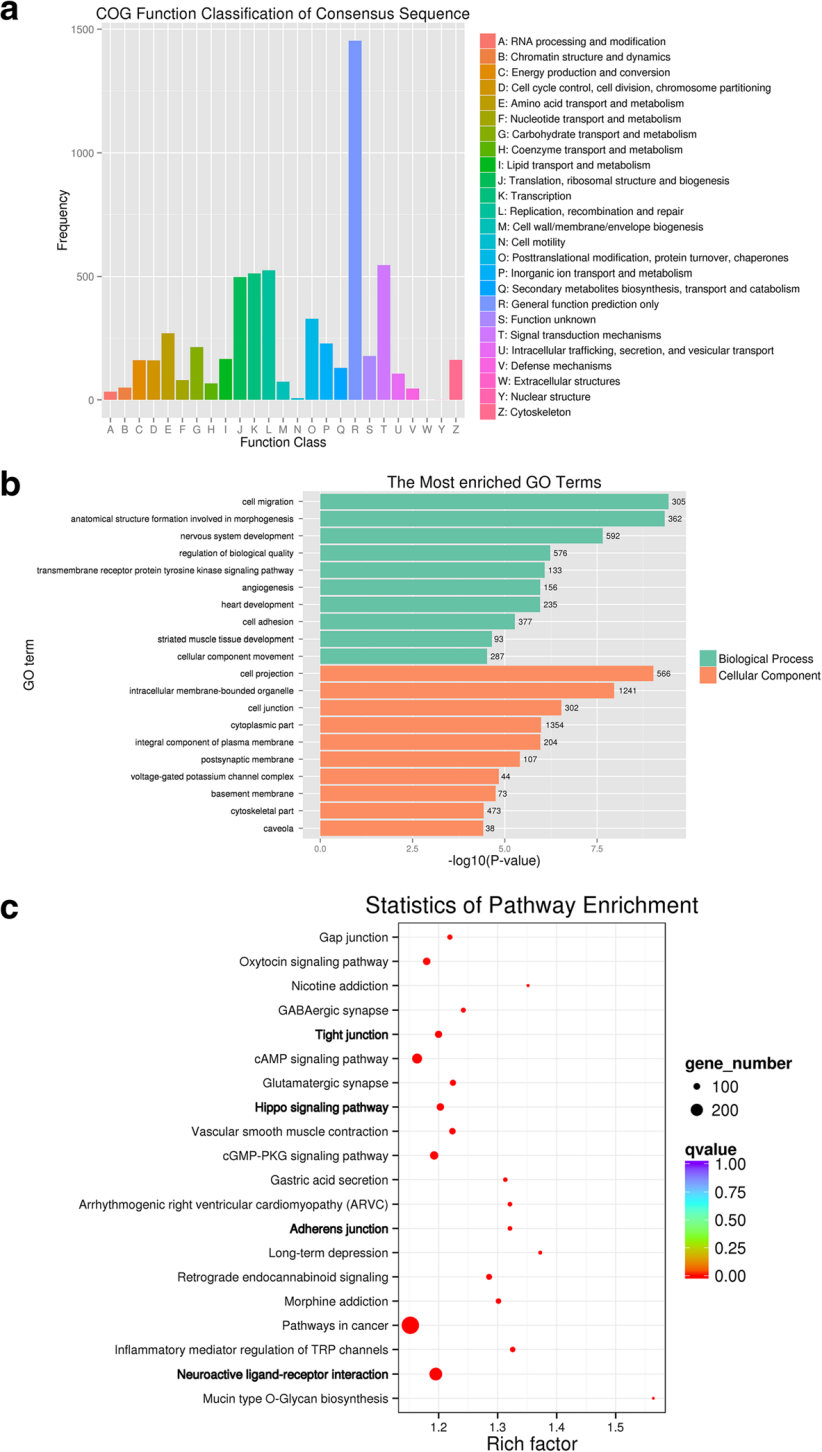
COG, top GO and top KEGG pathway analysis of CG type DMGs. a, COG analysis. b, top GO analysis. c, top KEGG analysis. In graph a, the abscissa represents the COG function classification; the ordinate represents the number of genes that were enriched in this classification. In graph b, the ordinate represents the GO terms that were the most enriched; the abscissa represents the *P*-value that was calculated using -log10-transformed values; the green and orange colors indicate biological process and cellular component. The size of the circles represent the number of genes contained in the particular class in the graph c, the larger the circle is, the more genes there are; Differently colored circles represent the enrichment degree of false positives, the redder the circle is, the lower is the false positive rate

To probe changes in the methylation status of gene functions under prolificacy traits, the COG, GO and KEGG pathway databases were analysed to characterize the 12,832 DMGs that were detected in the DMRs. The COG analyses revealed that DMGs were enriched on general function prediction mostly for CG (Fig. 10a). The GO analysis revealed that for the CG type, DMGs were significantly enriched in the categories of cell migration, anatomical structure formation involved in morphogenesis, cell projection, and intracellular membrane-bounded organelle (Fig. 10b). The KEGG analysis revealed that for CG type, DMGs were significantly enriched in the categories mucin type O-Glycanbiosynthesis, long-term depression and nicotine addiction (Fig. 10c). Importantly, we also found that some DMGs were involved in biological processes important for female gonad development, including ovarian follicle development (GO: 0001541), ovulation from ovarian follicle (GO:0001542), antral ovarian follicle growth (GO:0001547), luteinization (GO:0001553), ovulation cycle process (GO:0022602), negative regulation of female gonad development (GO:2,000,195), which suggested that these specific genes, which are influenced by DNA methylation could affect the development of ovarian follicles, subsequently impacting ewes’ prolificacy. For more detailed results of the COG, GO, KEGG analyses of CG, CHG and CHH (see Additional files 7, 8, 9 and 10).

### Correlation analysis of DMGs and sheep prolificacy

To further understand the relationship between DNA methylation and different levels of prolificacy, we set two limiting factors to perform an association analysis. First, the DMGs of the two groups should be enriched in female reproduction related pathways in the GO analysis. Second, the pathway in KEGG (except for disease and cancer pathways) that was enriched for the selected DMGs should significantly differ between the two groups (*P* < 0.05). As a result, 28 genes meeting these two criteria were detected (more detailed information on these genes is listed in Additional file 11: Table S7). Subsequently, these genes were analyzed using the STRING database.

**Fig. 11:**
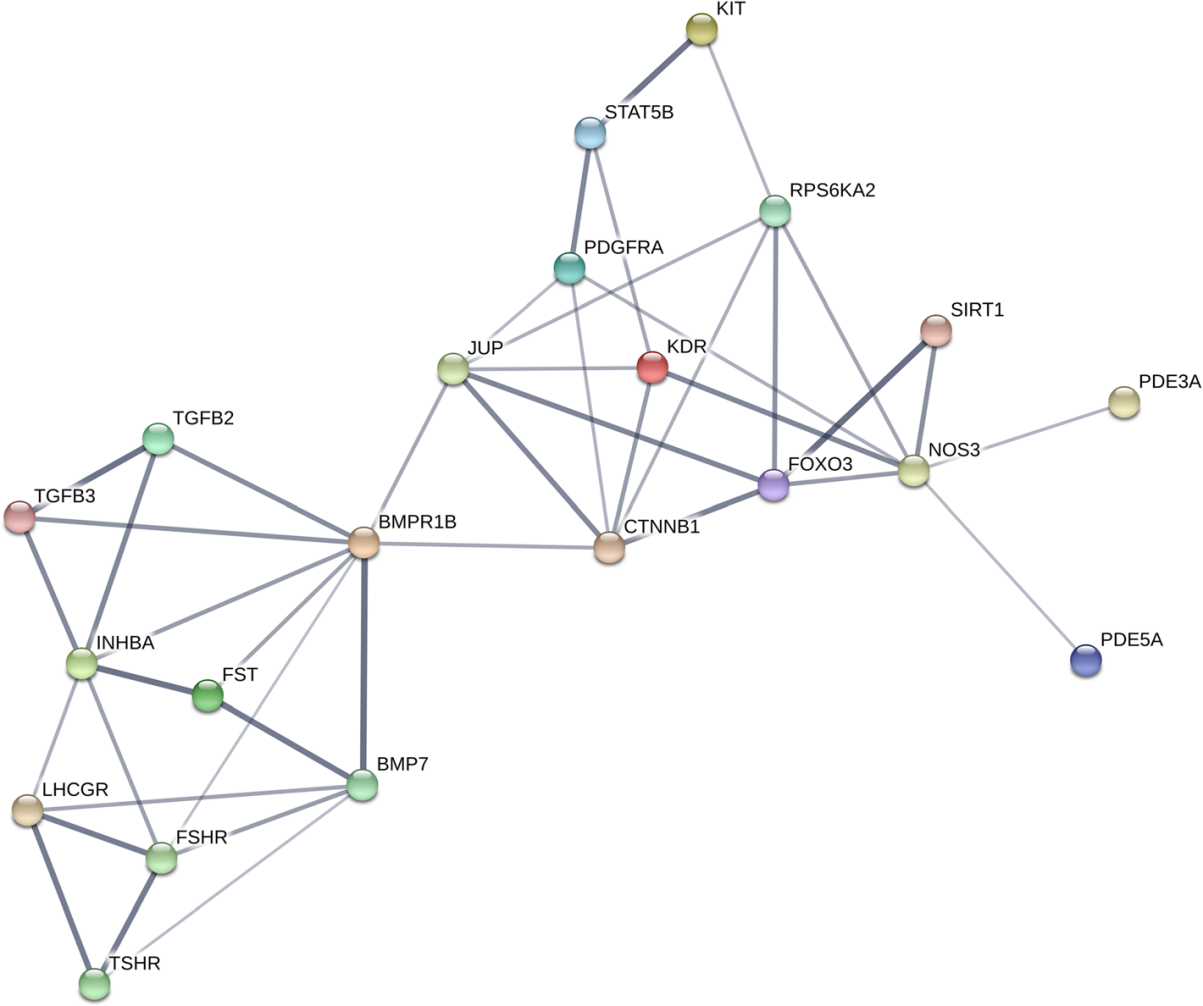
STRING analysis of DMGs associated with prolificacy. The detected DMGs were analyzed using the STRING database, with setting as follows: organism: *Bos Taurus*, interaction score: medium confidence, 1st shell: none/query proteins only, 2nd shell: none

As Fig. 11 shows, within the network analysis, we focused on the DMGs that interacted with 5 or more other genes. *BMP7*, *BMPR1B*, *CTNNB1*, *FST*, *FSHR*, *LHCGR*, *TGFB2*, and *TGFB3* are hub genes in the network, related to the female reproduction pathway. More detailed results on the abovementioned genes are listed in Table 2.

**Table 2:** DMGs most likely involved in female prolificacy pathways

Gene Name	DMR			GO pathway	GO ID	KEGG Pathway	KO ID
	location in chr	meth diff (LP vs HP)	*P*-value				
BMP7	13(58222103–58,222,185)	−0.243	4.71E-08	menstrual cycle phase	GO:0022601	TGF-beta signaling pathway	ko04350
	13(58258207–58,258,215)	0.203	8.29E-06			Hippo signaling pathway	ko04390
	13(58174539–58,174,596)	0.218	0.000243			Cytokine-cytokine receptor interaction	ko04060
	13(58235661–58,235,840)	0.344	3.01E-06				
BMPR1B	6(29427102–29,427,372)	−0.232	7.32E-05	ovarian cumulus expansion	GO:0001550	TGF-beta signaling pathway	ko04350
	6(29490707–29,490,892)	0.227	0.000308			Hippo signaling pathway	ko04390
	6(29297691–29,297,945)	0.266	6.21E-06			Cytokine-cytokine receptor interaction	ko04060
CTNNB1	19(14002501–14,002,561)	−0.541	2.32E-10	oocyte development	GO:0048599	Hippo signaling pathway	ko04390
	19(14008957–14,009,244)	−0.442	9.27E-14			Adherens junction	ko04520
	19(13978757–13,978,906)	−0.424	4.50E-20			Tight junction	ko04530
	19(13870396–13,870,492)	−0.409	9.06E-11			Leukocyte transendothelial migration	ko04670
	19(13826519–13,826,590)	−0.333	3.17E-10				
	19(13831621–13,831,667)	−0.322	1.81E-11				
	19(14018620–14,018,858)	−0.313	3.71E-07				
	19(13959030–13,959,259)	−0.308	2.10E-14				
	19(13989628–13,989,661)	−0.282	1.63E-06				
	19(13882120–13,882,179)	−0.275	8.06E-08				
	19(13959905–13,959,997)	−0.271	0.00168				
	19(13993527–13,994,032)	−0.243	5.13E-11				
	19(13685445–13,685,483)	−0.231	7.72E-05				
	19(13675322–13,675,759)	0.207	7.68E-07				
	19(13676205–13,676,451)	0.209	1.74E-05				
	19(13706582–13,706,880)	0.233	8.69E-07				
	19(13853573–13,853,684)	0.277	1.04E-07				
	19(13793737–13,793,755)	0.319	0.000111				
	19(13707639–13,707,973)	0.344	3.25E-07				
	19(13682987–13,683,047)	0.404	4.49E-08				
	19(13815477–13,815,573)	0.418	1.70E-06				
	19(13708400–13,708,447)	0.617	2.94E-26				
FSHR	3(75192698–75,192,705)	−0.371	2.39E-05	primary ovarian follicle growth	GO:0001545	cAMP signaling pathway	ko04024
	3(75379496–75,379,558)	−0.365	1.81E-08			Neuroactive ligand-receptor interaction	ko04080
	3(74644092–74,644,099)	0.154	8.92E-05				
	3(74716438–74,716,537)	0.213	9.40E-06				
	3(75040358–75,040,859)	0.218	9.66E-07				
	3(75387483–75,387,659)	0.224	0.000138				
	3(75366395–75,366,601)	0.226	5.29E-05				
	3(75298678–75,298,880)	0.249	3.49E-09				
	3(75134625–75,134,944)	0.257	6.08E-07				
	3(74739631–74,739,782)	0.352	3.21E-08				
	3(75132060–75,132,090)	0.402	9.62E-11				
	3(75027605–75,027,609)	0.466	1.72E-08				
	3(75289648–75,289,665)	0.483	2.63E-09				
	3(75291188–75,291,243)	0.625	9.79E-18				
FST	16(25647868–25,647,877)	−0.291	2.30E-06	female gonad development	GO:0008585	TGF-beta signaling pathway	ko04350
LHCGR	3(75688216–75,688,347)	−0.286	1.48E-05	ovarian follicle development	GO:0001541	Neuroactive ligand-receptor interaction	ko04080
	3(75736243–75,736,624)	−0.214	1.87E-07				
	3(75712912–75,712,941)	0.209	0.00544				
TGFB2	12(20040207–20,040,350)	0.229	5.70E-08	menstrual cycle phase	GO:0022601	Cytokine-cytokine receptor interaction	ko04060
	12(20044759–20,045,177)	0.23	2.80E-05			FoxO signaling pathway	ko04068
	12(20286676–20,286,930)	0.245	0.000418			TGF-beta signaling pathway	ko04350
	12(20300287–20,300,401)	0.316	1.90E-06			Hippo signaling pathway	ko04390
	12(19982100–19,982,518)	0.206	4.89E-09				
	12(19995176–19,995,216)	0.243	5.97E-07				
	12(20020000–20,020,214)	0.278	8.20E-11				
TGFB3	7(84205818–84,206,098)	−0.205	0.000768	menstrual cycle phase	GO:0022601	Cytokine-cytokine receptor interaction	ko04060
	7(84097027–84,097,093)	0.217	0.000423			FoxO signaling pathway	ko04068
	7(84139476–84,139,762)	0.227	8.22E-06			TGF-beta signaling pathway	ko04350
	7(84057073–84,057,143)	0.24	4.51E-05			Hippo signaling pathway	ko04390
	7(84105957–84,106,089)	0.262	0.000133				
INHBA	4:79,346,323–79,346,467	0.281	7.62E-06	ovarian follicle development	GO:0001541	TGF-beta signaling pathway	ko04350
	4:79,358,172–79,358,309	0.2	1.35E-07	positive regulation of ovulation	GO:0060279		
	4:79,364,290–79,364,421	0.223	1.30E-05				
	4:79,410,632–79,410,649	0.433	3.46E-09				
	4:79,467,732–79,468,003	0.286	0.000264				
	4:79,470,908–79,471,096	0.324	8.99E-07				
	4:79,511,040–79,511,072	−0.227	0.00129				
	4:79,522,334–79,522,542	0.553	1.75E-12				
	4:79,524,739–79,524,926	0.346	1.16E-08				
	4:79,544,088–79,544,103	−0.309	1.46E-09				
	4:79,554,774–79,554,784	0.334	4.59E-06				
	4:79,568,886–79,568,895	0.545	1.61E-08				
	4:79,580,604–79,580,858	0.643	6.50E-17				
	4:79,581,799–79,582,035	0.507	6.30E-12				
	4:79,595,470–79,595,497	0.625	1.59E-15				
	4:79,601,022–79,601,037	−0.129	9.26E-06				
	4:79,605,844–79,606,024	0.465	5.60E-14				
	4:79,658,524–79,658,656	−0.28	0.000333				
	4:79,658,703–79,658,930	0.206	0.000186				
	4:79,794,804–79,794,808	0.385	1.32E-09				
	4:79,818,557–79,818,672	0.266	6.96E-09				
	4:79,821,799–79,821,883	0.403	7.91E-08				
	4:79,823,656–79,823,864	0.47	1.76E-08				
	4:79,829,698–79,829,729	−0.222	0.0017				
	4:79,836,260–79,836,367	0.56	5.75E-09				
	4:79,968,621–79,968,690	−0.37	1.87E-09				
	4:80,049,783–80,050,296	−0.254	4.52E-14				
	4:80,063,788–80,063,855	−0.244	1.31E-06				
	4:80,085,854–80,085,996	−0.223	4.88E-05				
	4:80,150,315–80,150,500	0.274	2.37E-06				
	4:80,157,514–80,157,656	0.475	6.14E-13				
JUP	11(41441802–41,442,083)	−0.22	0.000192	oocyte development	GO:0048599		
	11(41458174–41,458,384)	0.254	8.93E-07				

*chr* chromosome, *DMR* different methylated regions, *meth diff* the difference in methylation levels between HP and LP (LP vs HP); a positive number means the methylation levels of this region in the HP group are higher than those in the LP group, and a negative number means that the methylation levels of this region in the HP group are lower than those in the LP group, *GO pathway* The name of the GO term, *GO ID* The ID of the GO term, *KEGG pathway* The name of the KEGG pathway, *KO ID* The ID of the KEGG pathway

## Discussion

DNA methylation is the main feature of the epigenetic regulatory mechanism that plays an important role in the regulation of gene expression. Recently, studies have been conducted to identify the genome-wide methylation profiles of farm animals [15–18]. Previously, some studies have been conducted to describe DNA methylation for sheep ovary [19–22], but few reports from the ovarian genome-wide methylation pattern [23]. WGBS, which allows unbiased genome wide DNA methylation profiling, has allowed us to investigate prolificacy related DNA methylation in unprecedented detail [9]. In this study, we used WGBS to investigate the DNA methylation profiles of the genome in ovarian tissues of high prolificacy and low prolificacy sheep to discover the relationship between DNA methylation and different levels of prolificacy. Further correlation analysis indicated that several DMR-related genes were most likely involved in Hu sheep prolificacy.

*DNMTs* are the writers of the epigenome. *DNMTs* constitute a highly conserved family of proteins in mammals, and there are 3 major *DNMTs*: *DNMT1*, *DNMT3a* and *DNMT3b*. *DNMT1* is a maintenance *DNMT*, while *DNMT3a* and *3b* are de novo *DNMTs*. Lynch et al. (2016) indicated that *DNMT3a* has broad downstream effects on the timing of the genomic control of reproductive function [24]. Our results showed that the mRNA expressions of *DNMT1* and *DNMT3a* in ovary tissue were significantly lower in the HP group than that in the LP group, which indicated that *DNMTs* may regulate the transcription of genes associated with sheep prolificacy. In our study, approximately 3.5% of cytosine sites were methylated, and the CG methylation type was present in the highest proportion and at the highest level in the genome. These results were similar to those found in other species, including humans and pigs [17, 25]. Non-CpG information was also obtained in the present study, CAG was the most common sequence motif in the CHG mC sites, as also found in other studies [26], and the sequence motif in CHH differed between the two groups. The methylation levels near the TSS were the lowest of all the gene functional regions, which was consistent with the results found in pig ovaries by RRBS [27]. Most hypermethylation CGI (over 68%) were located in distal intergenic regions, while only 1.5% were in UTR.

As the DNA methylation status of promoter and gene body regions could affect gene expression thorough changes in chromatin structure or transcription efficiency [28, 29], we compared the genome-wide methylation patterns of the HP and LP sheep to identify DMGs that may affect prolificacy. We identified 71,271 DMRs and 12, 831 genes related to these DMRs were predicted, and 68.60% of the DMRs were located in distal intergenic regions, but only 0.27% of the DMRs were located in UTRs, a finding that was also similar to the previous research in pig ovary tissues by RRBS [27]. To further validate the sequencing results, qRT-PCR was performed to detect the mRNA expression of 10 randomly selected DMGs, the expression patterns of which were consistent with the sequencing data. Because DMGs such as *ABCG2*, *mTOR*, *STK3*, *ACVR1* and *PSMD7* may contain two or more DMRs, these did not show significant differences between the HP and LP groups in our study.

In our study, 11,520 of the 12,831 DMGs were enriched in three categories, as determined by GO analysis: biological processes, molecular function and cellular components. Strict conditions were followed to select the most likely DMR related genes involved in the regulation of ovarian functions. Eventually, we identified 10 eligible DMGs, compared with LP group, *CTNNB1*, *FST*, *LHCGR*, and *TGFB3* were hypomethylated, and *BMP7*, *BMPR1B*, *FSHR*, *TGFB2*, *INHBA* and *JUP* were hypermethylated in the HP group. Moreover, the DMRs of these DMGs were all located in intron and distal intergenic regions in the genome. Recent evidence has shown that intragenic DNA methylation plays a role in the regulation of alternative splicing [30].

### Key DMGs in the TGF-β superfamily

Bone morphogenetic proteins, which belong to the transforming growth factor β (*TGFβ*) superfamily, are known to have effects on reproduction. A mutation in the *BMPR1B* gene, called *FecB*, was the first major gene associated with prolificacy in sheep [31, 32]. Previously, it was also reported that highly prolific Booroola sheep have a mutation in the intracellular kinase domain of *BMPR1B* (ALK-6) which is expressed in both oocytes and granulosa cells, and is associated with hyper prolificacy of these ewes [31]. *BMPR1B* has an additive effect on ovulation rates and litter size in several sheep breeds [4]. *BMP7* has been reported to have a significant role in ovarian folliculogenesis due to its expression from the time of committed follicles onward in rat thecal cells [33, 34], and *BMP7* was shown to have the function of down-regulating *StAR* and progesterone production in human granulosa-lutein cells [35]. However, the information regarding how the *TGFβ* family alters mammalian reproduction through DNA methylation is limited. Our findings in Hu sheep also support the earlier reports, in which significant differences were found in the levels of hypermethylation *BMPR1B* and *BMP7* genes in HP ovaries, and the pathways involving these genes were enriched in ovarian cumulus expansion and the menstrual cycle phase respectively. Moreover, using STRING analysis, we also found that *BMPR1B* was the hub of these reproduction related DMGs and correlated with *BMP7*, *FSHR* and *LHCGR*.

### Key DMGs in Gonadotropin hormone

In our study, several DMGs related to hormone function, such as *FSHR* and *FST*, showed significant differences in methylation levels (*FSHR* and *FST* are up-regulated, and *LHCGR* is down-regulated) in ovary tissue from the HP group compared with that from the LP group, which may influence mRNA expressions of these genes. Earlier studies provided strong evidence that ovarian follicles lacking FSH or FSH receptors fail to progress to a preovulatory stage, resulting in infertility [36], and expression of the *LHCGR* at relatively high levels in granulosa cells was required for preovulatory follicles to respond to the midcycle surge of *LH* that promotes ovulation, oocyte maturation, and corpus luteum formation [37]. The relatively higher levels of expression were found for the transcripts of *FSHR* and *LHCGR* across ovaries and ovarian follicles in *FecB* carrier ewes [33]. *FST* has been considered to play an important role in ovarian development in species such as mice and pigs [38, 39]. *FST* secreted by granulosa cells specifically inhibits *FSH* biosynthesis and secretion. However, *FST* expression patterns show significant divergence among species; in our study, only one DMR (chr16:25,647,868–25,647,877 on the antisense strand, strong hypomethylation in the HP group) was related to *FST* and located in the distal intergenic region after the TSS, which indicated that the expression of *FST* may be influenced by this DMRs, just as intragenic DNA methylation status can down-regulate *IGF2* gene expression in bovines [40]. Above all, changes in these gonadotropin receptor mRNA expression levels may determine follicular responses to gonadotropins thereby inducing the release of ovum.

### Key DMGs in folliculogenesis and ovulation

In our study, several DMGs relating to folliculogenesis and ovulation were identified, including *CTNNB1, INHBA* and *JUP*. Previous studies have shown that *CTNNB1* can facilitate FSH induced follicular growth and decreases follicle atresia, but that it negatively affects LH induced ovulation and luteinization in mice [41]. Moreover, it plays important roles in regulating patterning and morphogenesis that are related to adherent junctions and are required for gonadogenesis [42, 43], and similar results were also found in our study, moreover, the DMRs related to *CTNNB1* were located in distal intergenic and intron regions (2nd, 4th, 5th of 36). *INHBA* expression was stimulated by *BMP15* in granulosa cells from wild type ewes, and may play roles in the increased ovulation rates [44], and in our study, *INHBA* was correlated with *FST*, *LHCGR*, *FSHR*, *BMPR1B*, *TGFB2* and *TGFB3*. Up to now, several studies have reported on the function of *JUP* in ovarian cancers [45–49], but information regarding *JUP* function in mammal reproduction has been limited, our study supports *JUP* as being related to oocyte development, as identified GO analysis, and two DMRs (chr11:41,441,802–41,442,083 on the antisense strand, strong hypomethylation in the HP group; chr11:41,458,174–41,458,384 on the antisense strand, strong hypermethylation in the HP group) were related to *JUP* and located in distal intergenic and intron regions respectively.

In summary, this study provides a comprehensive analysis of the DNA methylation profiles of Hu sheep ovaries for HP and LP ewes. We identified DMRs and genes associated with these regions. Pathway and network analyses of these DMRs revealed several candidate genes that may affect ovarian function including gonadotropin, folliculogenesis and ovulation. We will validate those DMR-related genes from this study in different stages of follicles development in the future. The results of this study might therefore provide novel clues for deciphering the epigenetic mechanisms of sheep ovarian function and will likely contribute to improving reproductive capacity.

## Conclusion

This study revealed the global DNA methylation patterns of sheep ovaries associated with high and low prolificacy. We explained the differences in genomic DNA methylation between HP and LP sheep, and we observed that several DMRs/DMGs were most likely related to changes in Hu sheep prolificacy. Our results demonstrate that DNA methylation may contribute to a better understanding of epigenetic regulation in sheep reproductive capacity.

## Additional files

Additional file 1: Table S1. Primer information for qRT-PCR. (DOCX 22 kb)

Additional file 2: Figure S1. Plot of genome chromosome 5-methylcytosine map. A, CG type. B, CHG type. C, CHH type. H = A, C or T. The methylation levels of each window are described using colors (the redder the window is, higher is the methylation level; the greener the window is, the lower is the methylation level). HP (J07, J08, J09), High Prolificacy. LP (J10, J11, J12), Low Prolificacy. (PNG 14 mb)

Additional file 3: Table S2. The rates of methylcytosine in gene functional elements in the HP group and the LP group. (XLSX 10 kb)

Additional file 4: Table S3. DNA methylation levels in gene functional elements in the HP group and the LP group. (DOCX 22 kb)

Additional file 5: Table S4. Information of DMRs in HP and LP. methChr: the chromosome the DMR is located. start: the start site of the DMR island in the chromosome. end: the end site of the DMR in the chromosome. width: the length of the DMR. methDif: the different levels of DMR between the LP and the HP groups. Annotation: the type of DMR in gene functional elements. geneStrand: + is the sense strand, - is the antisense strand. methType: the methylation type of the DMR. geneId: the gene number which the DMR is related. (XLSX 4 mb).

Additional file 6: Table S5. Information about the methylation tendency of the chosen DMGs and the mRNA expression of the corresponding gene for the two groups. CGI_start: the start site of the CpG island in the chromosome. CGI_end: the end site of the CpG island in the chromosome. CGI_width: the length of the CpG island. strand: * the gene strand in which the DMR is located. methDif: the different levels of DMR between the LP and the HP groups. Annotation: the type of DMR in gene functional elements. geneStrand: + is the sense strand, - is the antisense strand. meth_direction: the direction of the methylation difference for DMRs (LP vs HP). exp_direction: the direction of the expression difference for DMGs (LP vs HP). (XLSX 14 kb)

Additional file 7: Figure S2. GO and KEGG pathway analysis in CG type DMGs. A, GO analysis. C, KEGG analysis. (PNG 4 mb)

Additional file 8: Figure S3. COG, GO and KEGG pathway analysis in CHG-type DMGs. A, COG analysis. B, GO analysis. C, top GO. D, KEGG analysis. (PNG 3 mb)

Additional file 9: Figure S4. COG, GO and KEGG pathway analysis in CHH-type DMGs. A, COG analysis. B, GO analysis. C, top GO. D, KEGG analysis. E, top KEGG. (PNG 5 mb)

Additional file 10: Table S6. Information about the COG, GO and KEGG analyses of DMGs. (XLSX 2 mb)

Additional file 11: Table S7. DMGs that were selected under strict conditions. (DOCX 24kb)

## Abbreviations

ACVR1: Activin A receptor type 1
BACH1: BTB domain and CNC homolog 1
BMP7: Bone morphogenetic protein 7 precursor
BMPR1B: Bone morphogenetic protein receptor type 1B
CDIPT: CDP-diacylglycerol--inositol 3-phosphatidyltransferase
CGI: CG island
chr: chromosome
COG: Cluster of orthologous groups of proteins
CTNNB1: Catenin (cadherin-associated protein), beta 1
DMGs: Differential methylated regions related genes
DMRs: Differential methylated regions
DNMT1: DNA methyltransferase 1
DNMT3a: DNA methyltransferase 3 alpha
DNMT3b: DNA methyltransferase 3 beta
DNMTs: DNA methyltransferases
ELK4: ETS transcription factor
FDR: False discovery rate
FSHR: Follicle-stimulating hormone receptor precursor
FST: Follistatin
GAPDH: Glyceraldehyde-3-phosphate dehydrogenase
GO: Gene ontology
GPNMB: Glycoprotein nmb
HP: High prolificacy group
IGF2: Insulin like growth factor 2
INHBA: Inhibin beta A subunit
JUP: Junction plakoglobin
KEGG: Kyoto encyclopedia of genes and genomes
LHCGR: Luteinizing hormone/choriogonadotropin receptor
LP: Low prolificacy group
mC: Methylated cytosine
mTOR: Mechanistic target of rapamycin
PSMD7: Proteasome 26S subunit: non-ATPase 7
qRT-PCR: Quantitative reverse transcriptase PCR
RRBS: Representation bisulfite sequencing
StAR: Proteasome 26S subunit, non-ATPase 7
STK3: Serine/threonine kinase 3
TGFB2: Transforming growth factor beta 2
TGFB3: Transforming growth factor beta 3
TGFβ: Transforming growth factor beta
TSS: Transcription start site
UTR: Untranslated regions
WGBS: Whole-genome bisulfite sequencing
