# Supplementary material for: Genome-wide analysis of DNA Methylation profiles on sheep ovaries associated with prolificacy using whole-genome Bisulfite sequencing
Source: BMC Genomics. 2017 Oct 2;18:759. doi: 10.1186/s12864-017-4068-9 (PMC5625832; doi:10.1186/s12864-017-4068-9)
Supplement: Supplementary file 1 — Primer information for qRT-PCR. (DOCX 22 kb) [file 12864_2017_4068_MOESM1_ESM.docx]

**Table S1:** Primer information for qRT-PCR

| Primer Names | Sequences (5’-3’) | | | Product Length(bp) | Gene Bank |
| --- | --- | --- | --- | --- | --- |
|  | **Forward** | | **Reverse** |  |  |
| GAPDH | GTCAAGGCAGAGAACGGGAA | | GGTTCACGCCCATCACAAAC | 232 | NM_001190390.1 |
| DNMT1 | CCTGACTCCACCTACGAAGACC | | TCTACTTGCTCCACCACGAACT | 128 | NM_001009473.1 |
| DNMT3A | AAGCAGGGCAAAGACCAGCATT | | AGCGAAGAGGTGGCGGATGA | 189 | XM_012166008.2 |
| DNMT3B | CGCAGATCAAGCTCACGACT | CGGTTGGAGGTACTGCTGTT | | 203 | XM_004014479.3 |
| ABCG2 | CAGGTCAGAGTGTGGTGTCC | GTTACGTTGAGTCCTGGGCA | | 208 | NM_001078657.1 |
| PSMD7 | AAACTAACGCATTGTGCCGC | GTCCGAAACCTGAGCTGTCA | | 151 | XM_004015106.2 |
| ELK4 | AAGCCACCAGTTGAACCTCC | GCCACTGATTCCATGTCCGT | | 266 | XM_012186950.2 |
| CDIPT | GGACTTCTGGACGCTTTGGA | TGACTTTCACTGCCTCGGAC | | 230 | XM_004020893.3 |
| ACVR1 | TCGACATTTGGGCCTTTGGA | CCAGGGAGGTTAATGTCGGG | | 207 | XM_012166008.2 |
| BACH1 | ATGCTCTCTCGGATCCTCCC | TAACTGTCACCTCTCCGGGC | | 244 | XM_015091726.1 |
| SCYL1 | CGCCCTTATTCAAGGTGGGT | TGAGGTTGGTCTCGTTCAGC | | 279 | XM_012117216.2 |
| CPNMB | ACCCTGGGTGATGATGCAAG | AAGGCGATCACAGTGACGAG | | 143 | XM_004007790.3 |
| mTOR | TGAACAGCGAGCACAAGGAGA | GCCAAGACACAGTAGCGGATG | | 199 | NM_001145455.1 |
| STK3 | CCTGCTGCTTCTGATACGGT | CCCCCTGAATGTAGCAGGTG | | 108 | XM_012100056.2 |
