# Supplementary figures and images for: Genome-wide analysis of DNA Methylation profiles on sheep ovaries associated with prolificacy using whole-genome Bisulfite sequencing
Source: BMC Genomics. 2017 Oct 2;18:759. doi: 10.1186/s12864-017-4068-9 (PMC5625832; doi:10.1186/s12864-017-4068-9)

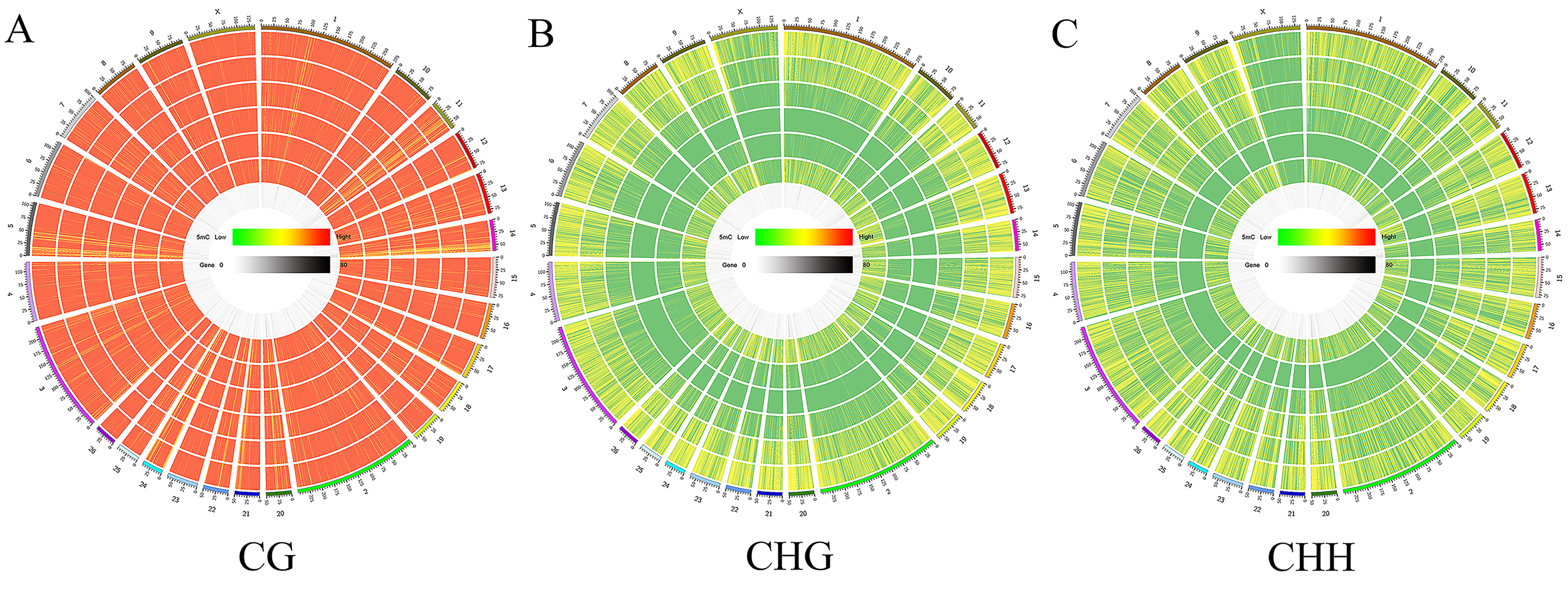

Supplement: Supplementary file 2 — Plot of genome chromosome 5-methylcytosine map. A, CG type. B, CHG type. C, CHH type. H = A, C or T. The methylation levels of each window are described using colors (the redder the window is, higher is the methylation level; the greener the window is, the lower is the methylation level). HP (J07, J08, J09), High Prolificacy. LP (J10, J11, J12), Low Prolificacy. (PNG 14 mb) [file 12864_2017_4068_MOESM2_ESM.png]

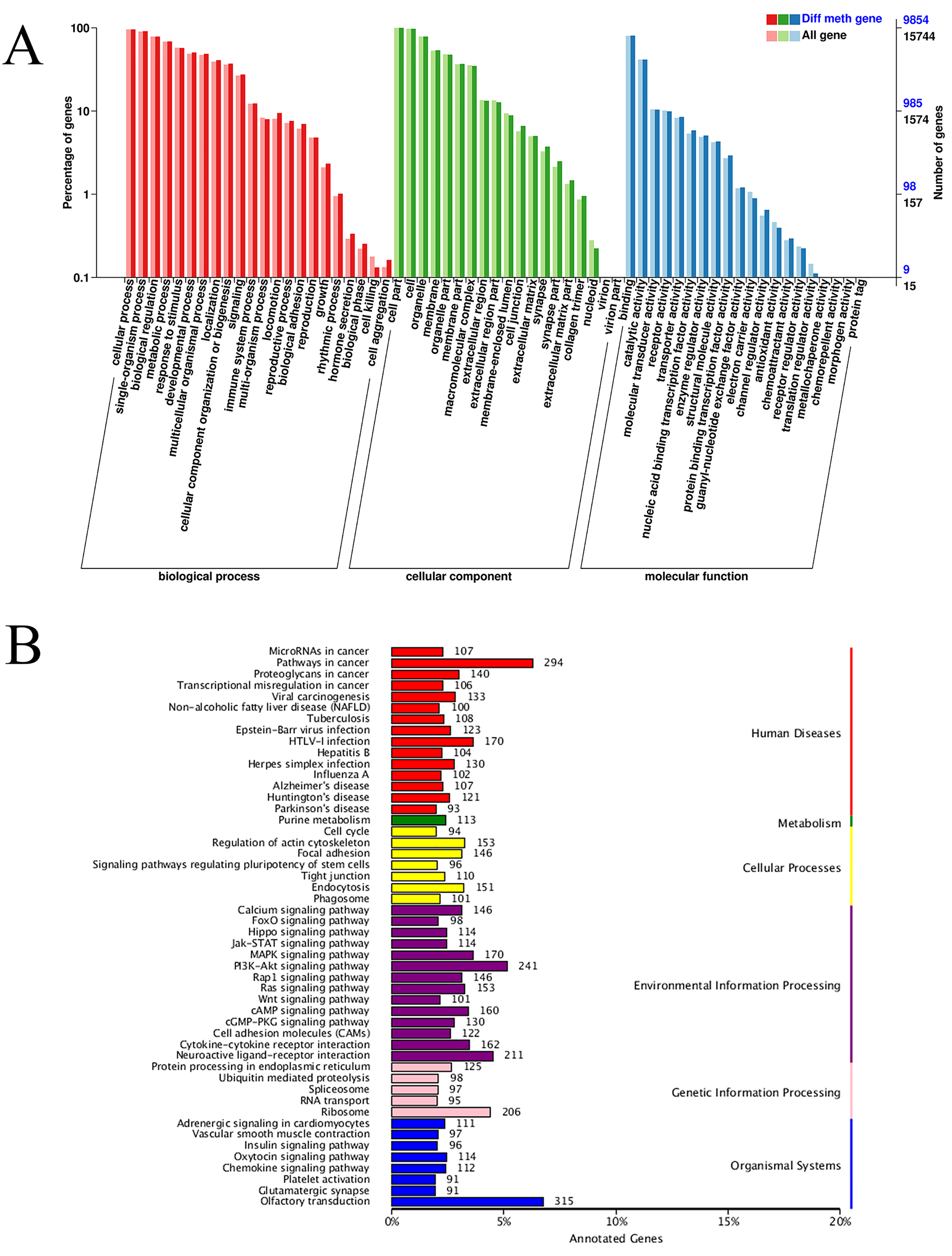

Supplement: Supplementary file 7 — GO and KEGG pathway analysis in CG type DMGs. A, GO analysis. C, KEGG analysis. (PNG 4 mb) [file 12864_2017_4068_MOESM7_ESM.png]

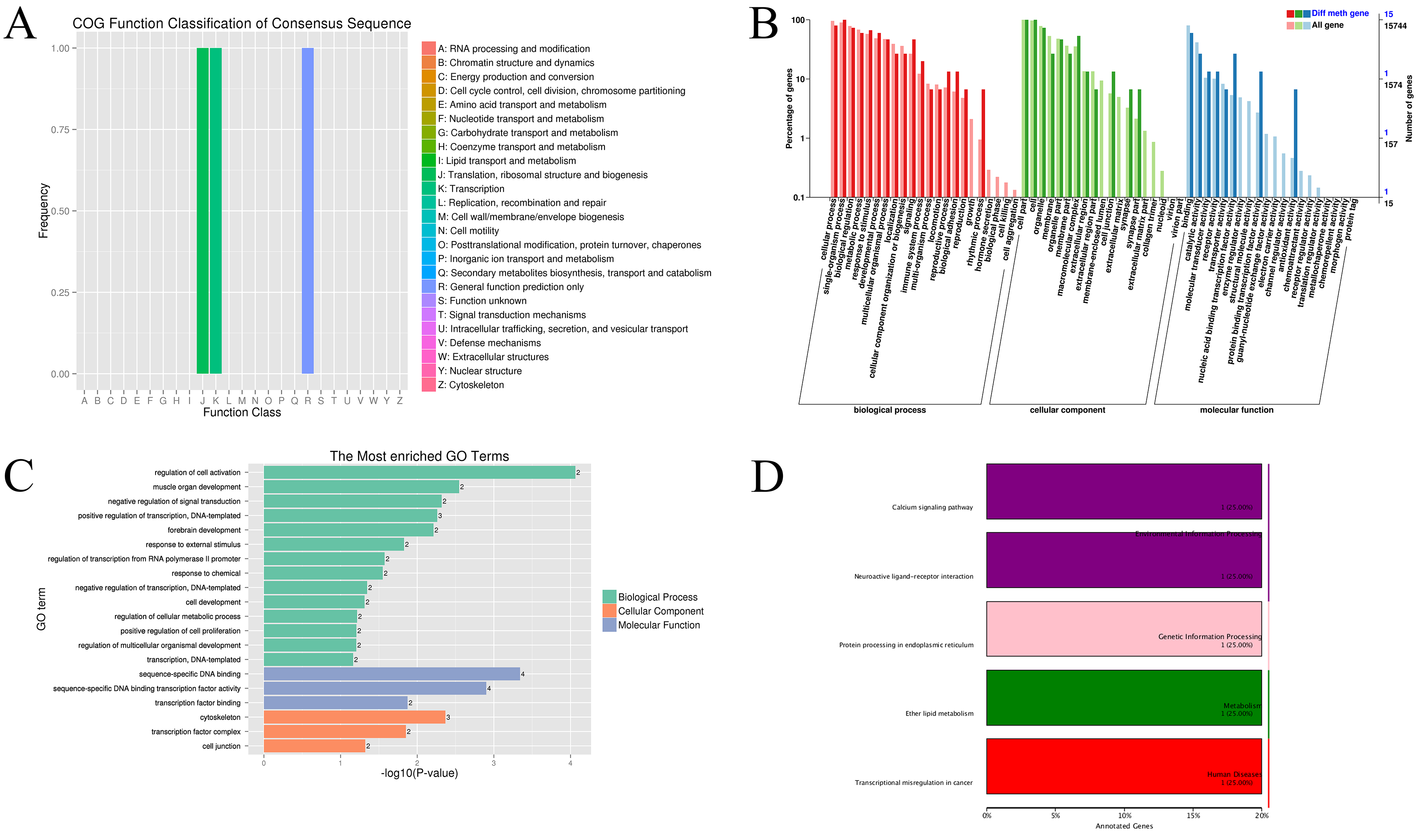

Supplement: Supplementary file 8 — COG, GO and KEGG pathway analysis in CHG-type DMGs. A, COG analysis. B, GO analysis. C, top GO. D, KEGG analysis. (PNG 3 mb) [file 12864_2017_4068_MOESM8_ESM.png]

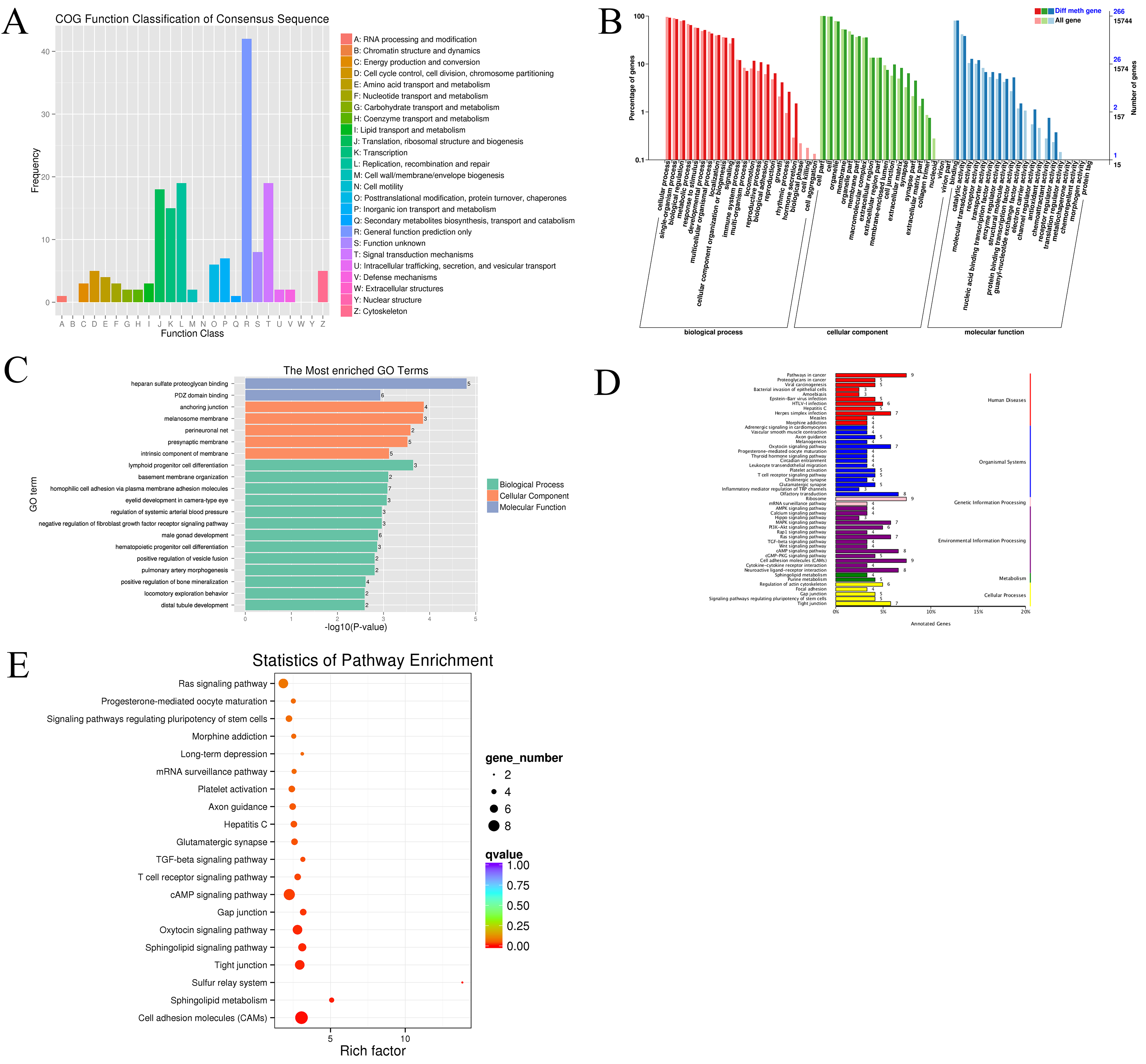

Supplement: Supplementary file 9 — COG, GO and KEGG pathway analysis in CHH-type DMGs. A, COG analysis. B, GO analysis. C, top GO. D, KEGG analysis. E, top KEGG. (PNG 5 mb) [file 12864_2017_4068_MOESM9_ESM.png]
