# Supplementary material for: Genome-wide analysis of DNA Methylation profiles on sheep ovaries associated with prolificacy using whole-genome Bisulfite sequencing
Source: BMC Genomics. 2017 Oct 2;18:759. doi: 10.1186/s12864-017-4068-9 (PMC5625832; doi:10.1186/s12864-017-4068-9)
Supplement: Supplementary file 4 — DNA methylation levels in gene functional elements in the HP group and the LP group. (DOCX 22 kb) [file 12864_2017_4068_MOESM4_ESM.docx]

**Table S3.** DNA methylation levels in gene functional elements in the HP group and the LP group.

| **Group** | **Context** | **upstream** | **first_exon** | **first_intron** | **inner_exon** | **inner_intron** | **last_exon** | **downstream** |
| --- | --- | --- | --- | --- | --- | --- | --- | --- |
| HP | CG | 0.455 | 0.3893 | 0.6923 | 0.7729 | 0.7522 | 0.7132 | 0.6676 |
|  | CHG | 0.0015 | 0.0015 | 0.0016 | 0.0016 | 0.0016 | 0.0016 | 0.0016 |
|  | CHH | 0.0014 | 0.0015 | 0.0016 | 0.0015 | 0.0016 | 0.0015 | 0.0015 |
| LP | CG | 0.4442 | 0.3805 | 0.6818 | 0.7675 | 0.744 | 0.7081 | 0.6605 |
|  | CHG | 0.0016 | 0.0016 | 0.0017 | 0.0017 | 0.0017 | 0.0017 | 0.0016 |
|  | CHH | 0.0015 | 0.0016 | 0.0017 | 0.0015 | 0.0017 | 0.0016 | 0.0015 |

**Note: The results use numbers to represent the average levels of DNA methylation in each gene functional element.**
