# Supplementary material for: Genome-wide analysis of DNA Methylation profiles on sheep ovaries associated with prolificacy using whole-genome Bisulfite sequencing
Source: BMC Genomics. 2017 Oct 2;18:759. doi: 10.1186/s12864-017-4068-9 (PMC5625832; doi:10.1186/s12864-017-4068-9)
Supplement: Supplementary file 11 — DMGs that were selected under strict conditions. (DOCX 24kb) [file 12864_2017_4068_MOESM11_ESM.docx]

**Table S7.** DMGs that were selected under strict conditions.

| Gene ID | Gene Name | Description |
| --- | --- | --- |
| ENSOARG00000018039 | BMP7 | bone morphogenetic protein 7 precursor |
| ENSOARG00000017161 | BMPR1B | bone morphogenetic protein receptor type 1B |
| ENSOARG00000002885 | CTNNB1 | catenin (cadherin-associated protein), beta 1 |
| ENSOARG00000004940 | FOXG1 | forkhead box G1 |
| ENSOARG00000010741 | FOXO3 | forkhead box O3 |
| ENSOARG00000004340 | FSHR | follicle-stimulating hormone receptor precursor |
| ENSOARG00000008119 | FST | follistatin |
| ENSOARG00000006474 | GLRB | glycine receptor beta |
| ENSOARG00000017475 | INHBA | inhibin beta A subunit |
| ENSOARG00000017474 | JUP | junction plakoglobin |
| ENSOARG00000001060 | KDR | vascular endothelial growth factor receptor 2 precursor |
| ENSOARG00000000078 | KIT | v-kit Hardy-Zuckerman 4 feline sarcoma viral oncogene homolog precursor |
| ENSOARG00000004455 | LHCGR | lutropin-choriogonadotropic hormone receptor |
| ENSOARG00000001979 | NOS3 | nitric oxide synthase 3 |
| ENSOARG00000020451 | PDE3A | phosphodiesterase 3A |
| ENSOARG00000016017 | PDE5A | phosphodiesterase 5A |
| ENSOARG00000019070 | PDGFRA | platelet derived growth factor receptor alpha |
| ENSOARG00000004895 | RPS6KA2 | ribosomal protein S6 kinase A2 |
| ENSOARG00000004826 | SIRT1 | sirtuin 1 |
| ENSOARG00000018964 | STAT5B | signal transducer and activator of transcription 5B |
| ENSOARG00000011583 | TGFB2 | transforming growth factor beta 2 |
| ENSOARG00000002050 | TGFB3 | transforming growth factor beta 3 |
| ENSOARG00000002882 | TSHR | thyroid stimulating hormone receptor |
| ENSOARG00000000130 | Novel gene | Novel gene |
| ENSOARG00000001262 | Novel gene | BCL2L1 (NCBI gene record; description: BCL2-like 1) |
| ENSOARG00000008334 | Novel gene | VEGFA (NCBI gene record; description: vascular endothelial growth factor A) |
| ENSOARG00000019122 | Novel gene | Novel gene |
| ENSOARG00000020129 | Novel gene | Novel gene |
